# Is acute kidney injury age-dependent in older adults: an observational study in two centers from North China

**DOI:** 10.1186/s12877-020-01906-z

**Published:** 2021-01-06

**Authors:** Libin Xu, Yanhua Wu, Yuanhan Chen, Ruiying Li, Zhiqiang Wang, Zhilian Li, Guoping Liu, Lei Yu, Wei Shi, Xinling Liang, on behalf of China collaborative study on AKI (CCS-AKI)

**Affiliations:** 1 Division of Nephrology, Guangdong Provincial People’s Hospital, Guangdong Academy of Medical Sciences, 106 Zhongshan Road 2, Guangzhou, 510080 China; 2 Department of Nephrology, Inner Mongolia People’s Hospital, Hohhot, Inner Mongolia Autonomous Region China; 3 Department of Nephrology, Hohhot First Hospital, Hohhot, Inner Mongolia Autonomous Region China

**Keywords:** Geriatric acute kidney injury, Older adults, Mortality, Creatinine, Epidemiological study, Hospitalized population

## Abstract

**Background:**

Although aging increases susceptibility to acute kidney injury (AKI), whether the AKI risk and the association between AKI and adverse outcomes are age-dependent remain unclear in older adults. The current study aimed to identify whether AKI risk was age-dependent in older adults and to investigate whether the association between AKI and mortality increased with increasing age.

**Methods:**

Medical records from 47,012 adult hospital admissions, including 30,194 older adults aged 60 or older, in two tertiary general hospitals were studied retrospectively. AKI was identified based on changes in blood creatinine levels according to the Kidney Disease: Improving Global Outcomes criteria.

**Results:**

Among the total population and 30,194 older adult patients, the raw incidences of AKI were 8.2 and 8.3%, respectively. The curve of the age-grouped AKI incidence was “U-shaped”, which revealed a positive relationship between the AKI incidence and age among the older adults aged 75 years or older. This trend of the age-AKI relationship was supported by further multivariable analysis. After adjusting for the Charlson Comorbidity Index score, the AKI was associated with in-hospital mortality; however, the associations did not increase with increasing age.

**Conclusion:**

The AKI risk does not increase with age in older adults, except for those aged 75 and above. The association between AKI and in-hospital death did not increase in an age-dependent manner in older adults.

**Trial registration:**

This study was retrospectively registered at clinicaltrials.gov (NCT03054142) on February 13, 2017.

## Background

Population aging is an increasing global problem; China’s aging population is increasing rapidly, and in 2016, the number of Chinese people > 65 years soared to 150 million, or 10.8% of the population [1]. This aging population has imposed heavy burdens on healthcare systems and has become a challenge for clinicians.

Age-related changes in kidney function and multiple comorbidities can increase the susceptibility of older adults to acute kidney injury (AKI) [2, 3]. AKI refers to an abrupt decrease in kidney function, previously termed acute renal failure, which is based typically accompanied by an elevation in the serum creatinine concentration [4, 5]. The change in terminology from acute renal failure to AKI reflects the recognition that smaller decreases in kidney function without overt organ failure are of substantial clinical relevance and are associated with increased morbidity and mortality [5]. The pooled incidence of hospital admissions according to the Kidney Disease: Improving Global Outcomes (KDIGO) definition was 21% in a preliminary meta-analysis including 266 studies (4,502,158 patients) [6]. Because AKI is strongly associated with increased morbidity and mortality, it has become an increasing global concern. However, the clinical characteristics and outcomes have not been well studied in older adults.

Several earlier studies have shown that older patients have a higher risk of acute renal failure or hospital-acquired renal insufficiency than younger patients [7, 8]. Some studies evaluating AKI among older adults included only older adults but did not compare this population with a non-older population [9, 10]. Even in a recent nationwide large-sample study, subjects were not stratified by age [11]. Thus, whether AKI in older adults is age-dependent and whether a cutoff age exists remain unclear.

Although the majority of studies have shown the adverse outcomes of AKI, whether it is age-dependent in older adults has not been demonstrated, and a few studies have indicated a negative conclusion. A previous study showed that older patients with acute renal failure in advanced age subgroups did not have a greater risk of mortality than those aged < 65 years [8]. In 82 patients with acute renal failure following cardiac surgery who required dialysis, the outcomes among older adults were comparable to those among the younger patients [12]. In older patients who underwent major surgery, a higher postoperative AKI grade was associated with increased in-hospital mortality only in patients ≤76 years and not in patients > 76 years [13, 14]. However, the sample sizes were very limited, and some important confounding factors were uncontrolled in these studies.

In the aforementioned context, we investigated whether the AKI risk was age-dependent in older adults and explored whether the association between AKI and mortality increased with increasing age. To achieve these aims, a large-sample investigation was conducted in two tertiary centers in China.

## Methods

### Study design and data source

A retrospective investigation was conducted using electronic medical records from Inner Mongolia People’s Hospital (IMPH) and Hohhot First Hospital (HFH). IMPH (3000 beds) and HFH (1200 beds) are both tertiary general hospitals located in Hohhot, the capital of the Inner Mongolia Autonomous Region of Northern China. Data were derived from the China Collaborative Study on AKI (CCS-AKI), which was sponsored by the Guangdong Provincial People’s Hospital (GPPH). This study was registered at clinicaltrials.gov on 2017 February 13 (NCT03054142). The multicenter study protocol complied with the Declaration of Helsinki and was approved by the Ethics Research Committee of Guangdong Provincial People’s Hospital (GDREC2016327H). It was also approved by the Ethics Research Committee of Hohhot First Hospital (20170210) and the Ethics Research Committee of Inner Mongolia People’s Hospital (20160825). These committees waived the normal requirement for informed consent because we only worked with deidentified records and linked data.

### Subjects

All patients aged 18 years or older who were admitted to IMPH between February 2012 and September 2016 and to HFH between February 2012 and December 2016 were screened. A total of 56,101 admissions with the necessary medical records were enrolled.

Patients who were not applicable for AKI evaluation were excluded from the subsequent risk factor analysis. These conditions were as follows: lacking at least 2 creatinine tests during hospitalization, previous amputations, advanced chronic kidney disease (CKD) and extremely low creatinine values (peak creatinine < 0.6 mg/dl). CKD was classified by the estimated glomerular filtration rate, which was calculated based on the minimum creatinine level during the hospitalization by the CKD Epidemiology Collaboration creatinine equation [15]. The definition of advanced CKD included the following: (1) a diagnosis record of stage 5 CKD or equivalent diagnosis, (2) a glomerular filtration rate less than 15 ml/min/1.73 m^2^, or (3) a minimum serum creatinine more than 4.0 mg/dL.

### Identification and classification of AKI

AKI was defined as an increase in serum creatinine of 0.3 mg/dL within 48 h or a 50% increase in creatinine from baseline within 7 d based on the KDIGO criteria [16]. To calibrate the intrahospital difference in creatinine measurements, 20 samples were tested for creatinine (ranging from 0.5 to 10 mg/dL) in each of the two study centers and GPPH. The calibrated values, which were based on the linear regression coefficients with GPPH, were used in this study. To screen for AKI, blood creatinine data across all hospitalizations were sorted in both increasing and decreasing order according to the time of testing. According to the KDIGO-AKI definition, AKI can be further categorized into the community-acquired subtype and the hospital-acquired subtype, according to whether AKI occurs within 48 h after admission. However, because preadmission creatinine was unavailable in the dataset of the current study, the type of AKI could not be identified accurately. Therefore, we did not analyze AKI by subtype. The stage of AKI was determined using the peak creatinine level after AKI onset.

### Determining comorbidities

The International Classification of Disease (ICD) Code had not been standardized, and ICD codes were not identical to the diagnostic records in local hospitals; thus, comorbidities were screened using key fields from the electronic diagnostic records and then confirmed by trained nephrologists. Due to the lack of available resources, we only screened the fields associated with dialysis, hypertension and items included in the Charlson Comorbidity Index. Mortality was based on the death information in the electronic medical records.

### Statistical analyses

Continuous variables are presented as medians (25th and 75th percentiles), and categorical data are presented as percentages. The raw incidence of AKI was calculated with a formula (number that met the AKI criteria – number that were ineligible for AKI evaluation)/all admissions, as described previously [11]. Variables were entered into a multivariate logistic regression model by the forward logistic regression (LR) method, and their associated 95% confidence intervals (95% CIs) were estimated. To focus on the effect of age, an interaction effect was tested with age stratification. If a significant interaction was detected between age and another variable, separate models for different age subgroups were generated. All statistical analyses were performed using IBM SPSS 24.0 (Armonk, NY, USA), and a two-tailed *P* <  0.05 was considered statistically significant.

## Results

### Prevalence of detected AKI

Among all 56,101 hospital admissions during the study period, 47,012 patients aged 18 years or older and 30,194 patients aged 60 years or older met the inclusion criteria. In the 47,012 included admissions, 3846 (8.2%) met the criteria for AKI. The numbers of admissions with grades 1, 2 and 3 AKI were 3055 (6.5%), 555 (1.2%) and 236 (0.50%), respectively. Table 1 shows the clinical characteristics of the enrolled admissions without or with AKI. In the 30,194 older adult patients, 2509 had AKI, and the raw incidence of AKI was 8.3%, which was similar to that of the total population.

**Table 1:** Clinical characteristics of the study subjects

	Non-AKI (*n* = 43,166)	AKI (*n* = 3846)	*P* value
Age (years)	64 ± 16	65 ± 18	0.005
Male [n (%)]	26,053 (60.4%)	2184 (56.8%)	< 0.001
eGFR (ml/min/1.73 m^2^)	87.1 ± 24.9	86.9 ± 36.4	0.653
Length of hospital stay (days)	15 (11, 23)	17 (10, 31)	< 0.001
**Comorbidity** [n (%)]			
Hypertension	12,064 (27.9%)	973 (25.3%)	< 0.001
Myocardial infarction	1987 (4.6%)	232 (6.0%)	< 0.001
Congestive heart failure	6983 (16.2%)	560 (14.6%)	0.009
Peripheral disease	7795 (18.1%)	744 (19.3%)	0.047
Cerebrovascular disease	7795 (18.1%)	744 (19.3%)	0.047
Chronic pulmonary disease	6019 (13.9%)	364 (9.5%)	< 0.001
Dementia	173 (0.4%)	16 (0.4%)	0.886
Connective tissue disease	703 (1.6%)	68 (1.8%)	0.514
Peptic ulcer disease	517 (1.2%)	44 (1.1%)	0.769
Mild liver disease	5134 (11.9%)	496 (12.9%)	0.066
Diabetes without end-organ damage	5428 (12.6%)	460 (12.0%)	0.270
Hemiplegia	49 (0.1%)	0 (0.0%)	0.037
Mild to moderate renal disease	3248 (7.5%)	432 (11.2%)	< 0.001
Diabetes with end-organ damage	1946 (4.5%)	154 (4.0%)	0.147
Tumor without metastasis	8702 (20.2%)	682 (17.7%)	< 0.001
Leukemia	232 (0.5%)	27 (0.7%)	0.186
Lymphoma	87 (0.2%)	18 (0.3%)	< 0.001
Moderate or severe liver disease	229 (0.5%)	39 (1.0%)	< 0.001
Metastatic solid tumor	1572 (3.6%)	164 (4.3%)	0.050
Charlson Comorbidity Index	2 (0, 3)	2 (0, 3)	0.955
**Medications** [n (%)]			
Aminoglycosides	4310 (10.0%)	637 (16.6%)	< 0.001
Glycopeptides	191 (0.4%)	69 (1.8%)	< 0.001
ACEIs or ARBs	7412 (17.2%)	610 (15.9%)	0.038
Diuretics^a^	6336 (14.7%)	1070 (27.8%)	< 0.001
NSAIDs	4810 (11.1%)	675 (17.6%)	< 0.001

*eGFR* estimated glomerular filtration rate, *ACEI* angiotensin-converting enzyme inhibitor, *ARB* angiotensin II receptor antagonist

^a^ including antihypertensive drug combination

### Relationship between age and AKI

**Fig. 1:**
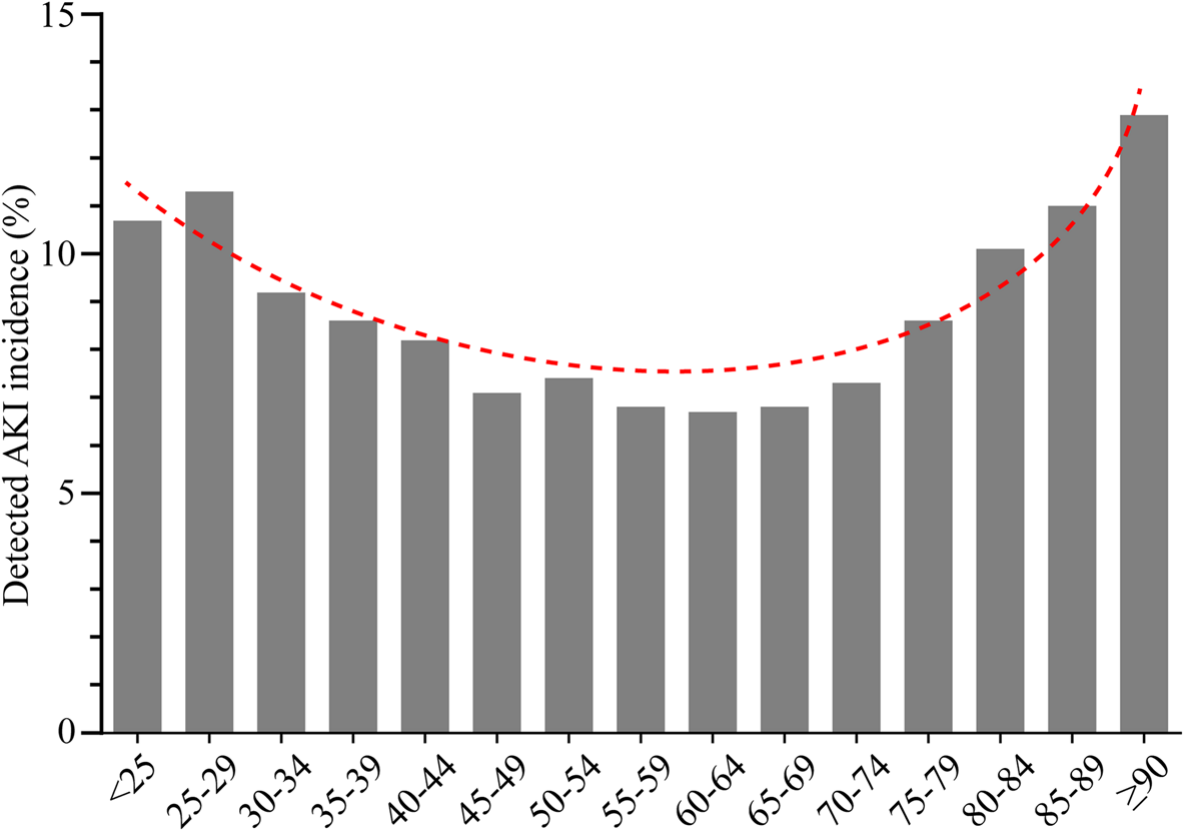
The raw incidence of AKI along an age gradient

We investigated the AKI incidence by age subgroup with 5-year intervals. The two age groups on the ends of the spectrum were 18–24 years and 90 years or older. The curve of total AKI incidence was “U shaped”, and the incidence was lowest in the age group of 45–74 years (Fig. 1).

**Fig. 2:**
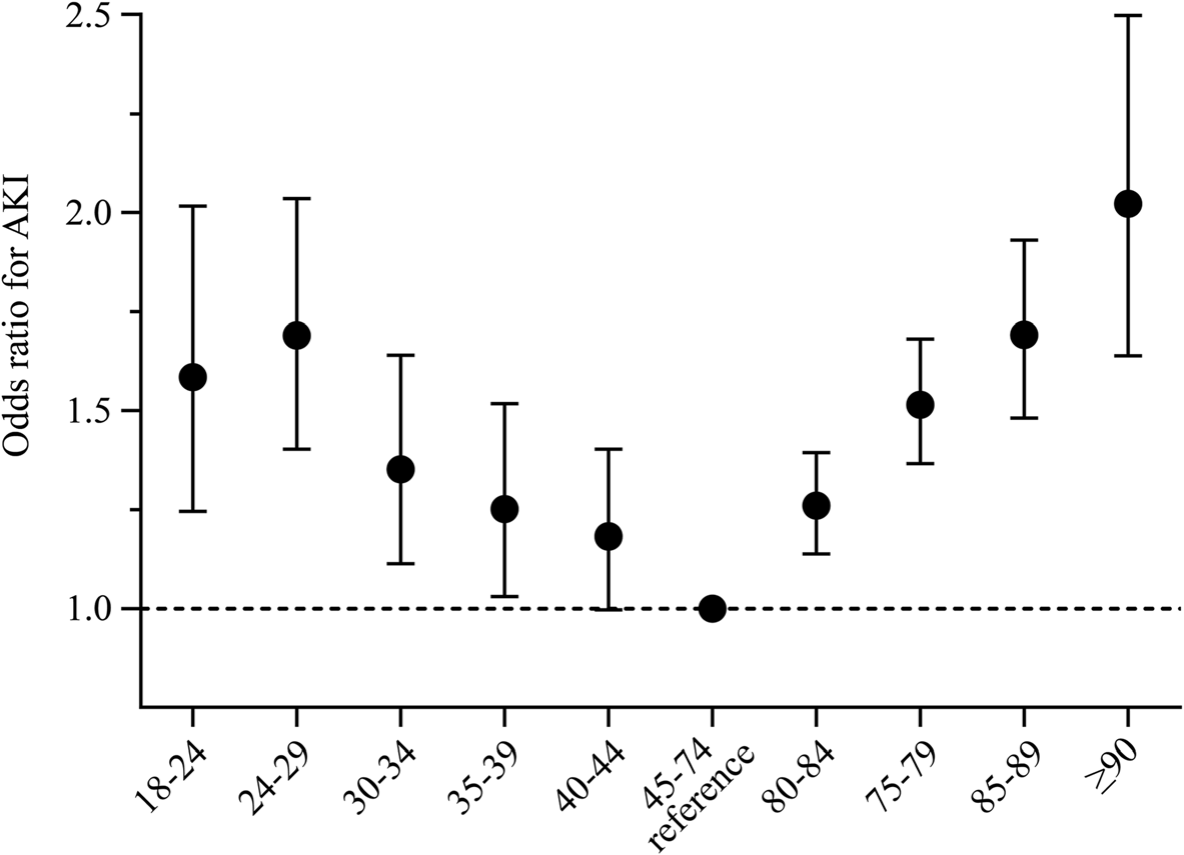
Risk of AKI stratified by age Black dots and error bars denote the odds ratios of the risk of AKI and its 95% confidence intervals compared with the reference age, 45–74 years. The dashed line denotes an odds ratio of 1.0. Included variables selected into the multivariable logistic regression model by the forward logistic regression (LR) method were eGFR stratification, hypertension, diabetes mellitus, myocardial infarction, peripheral angiopathy, cerebrovascular disease and heart failure.

We further tested the association between the AKI risk and age in multivariable logistic regression models. We defined 45–74 years as a reference age for AKI based on the lowest AKI incidence in univariable analysis. After adjusting for eGFR, hypertension, diabetes mellitus, myocardial infarction, peripheral angiopathy, cerebrovascular disease and heart failure, the risk of AKI was increased in the age subgroups younger than 45 years or older than 75 years compared with the reference age group (Fig. 2).

### AKI-related and age-related in-hospital mortality

**Fig. 3:**
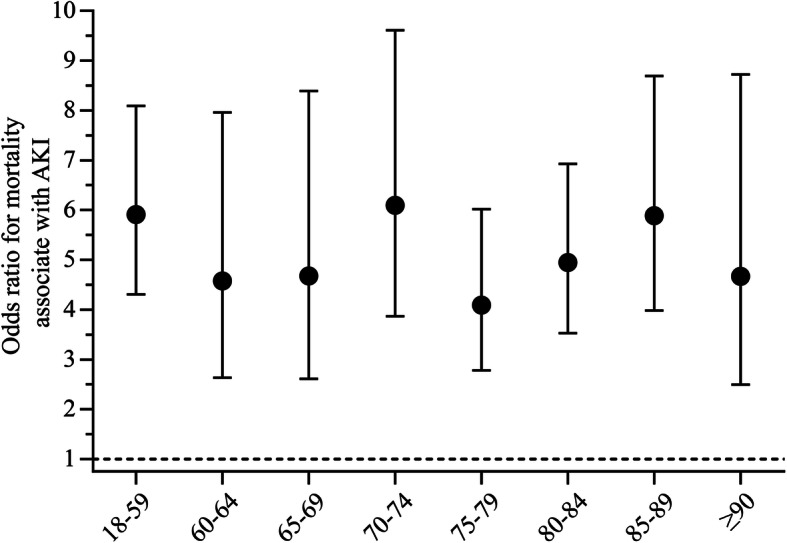
Risk of in-hospital mortality in the older adults stratified by age Black dots and error bars denote the odds ratios and 95% confidence intervals of the mortality risk for patients with AKI compared with non-AKI patients. The dashed line denotes an odds ratio of 1.0. The mortality risk associated with AKI was adjusted for the Charlson Comorbidity Index.

To investigate the clinical outcomes of AKI in the older adults, we studied the relationship between AKI and in-hospital mortality. Because of the interaction between AKI and age group in the preliminary analysis, we analyzed the relation of AKI and in-hospital mortality stratified by age group with 5-year intervals. After adjusting for the Charlson Comorbidity Index score, AKI was still associated with a higher risk for in-hospital mortality in each age group. These associations did not increase with age, showing an age-independent trend (Fig. 3).

## Discussion

The “U shaped” distribution revealed a positive relationship of AKI incidence and age among the older adults aged 75 years or older. Further multivariable analysis confirmed this cut-off age for higher risk of AKI. We further studied the association between AKI and mortality stratified by age. After adjusting for the Charlson Comorbidity Index scores, the AKI was associated with in-hospital death in all age subgroups in the older adults; however, these associations were not age-dependent.

Aging is regarded as an important risk factor for AKI. The AKI risk-age subgroup distribution curves indicated that the relatively younger adults does not have any additional risk for AKI. Although we could not compare the AKI risk stratified by age with one-year intervals due to limited sample size, the observation of this trend will facilitate the investigation of the cut-off age in older adults in future studies.

The 8.2% raw incidence of detected AKI in the hospitalized population of this study was much lower than the 21% pooled AKI prevalence in a previous report [6], which might be attributed for several reasons. First, AKI classification was based on the short-term changes in creatinine and urine output. Because of the retrospective nature, urine volume records were not available, which caused the underestimation of AKI. In addition, the preadmission level of creatinine was unavailable in our electronic records, which could result in the underestimation of AKI. Third, the majority of epidemiological studies on AKI have focused on patients in a critical condition and patients with cardiovascular disease; however, this study involved a general hospitalized population. The clinical characteristics and criteria for admission and discharge might differ from those in other studies that focused on specific populations; for example, the length of stay was relatively longer in our study (Table 1). Thus, our results revealed the epidemiological features of the older adults in a general hospitalized population.

There are several limitations due to the retrospective nature of this study. First, there is a low frequency of performance of the creatinine test in China [17]; therefore, only the patients with repeated creatinine tests during hospitalization were included to reduce the effect of fewer creatinine tests. Among all the admissions, 16% that were not evaluated for AKI were further excluded. Thus, selection bias could not be avoided. Second, due to the lack of time stamps on the diagnosis and medication data, we could not identify causal relations among acute morbidities, medications and AKI. Thus, the cause of AKI could not be investigated. Furthermore, although we standardized the ICD-10 codes relative to the Charlson Comorbidity Index, other codes were not standardized between hospitals; thus, other comorbidities could not be controlled for as confounding factors. In addition, several important factors, such as interventions, were lacking in the dataset.

## Conclusion

The AKI risk did not increase with increasing age in older adults, except for the patients aged 75 and above. In addition, although AKI is associated with in-hospital death in older adults, older patients with AKI did not have a higher risk for mortality than the relatively younger adults.

## Data Availability

The datasets used and/or analysed during the current study are available from the corresponding author on reasonable request.

## Abbreviations

AKI: acute kidney injury
CI: confidence interval
GPPH: Guangdong Provincial People’s Hospital
KDIGO: Kidney Disease: Improving Global Outcomes
IMPH: Inner Mongolia People’s Hospital
HFH: Hohhot First Hospital
CCS-AKI: China Collaborative Study on AKI
ICD: International Classification of Disease
CKD: chronic kidney disease
